# Assessing the accumulated stickiness magnitude from fabric–skin friction: effect of wetness level of various fabrics

**DOI:** 10.1098/rsos.180860

**Published:** 2018-08-22

**Authors:** Ka-Po Maggie Tang, Kam-Hong Chau, Chi-Wai Kan, Jin-tu Fan

**Affiliations:** 1 Institute of Textiles and Clothing, The Hong Kong Polytechnic University, Hung Hom, Hong Kong; 2 College of Human Ecology, Cornell University, Ithaca, NY 14853, USA

**Keywords:** adhesion, stickiness sensation, friction, magnitude estimate, fabric, wet skin

## Abstract

Increasing skin wetness tends to increase fabric–skin adhesion and friction, resulting in wear discomfort or skin injuries. Here, the magnitude estimation approach was used to assess the stickiness sensation perceived in fabrics. Seven fabric types were wetted by putting onto wet ‘skin’ surface and dried for different durations to achieve different wetness levels, simulating wearing conditions during the recovery period after sweating. Results showed that the relationship between magnitude estimates of stickiness and amount of water present in fabric demonstrated a power function. The exponents and constant from power regression show the growth rate of stickiness sensation with moisture intensity and the perceived stickiness under fixed stimulus intensity, respectively. A novel parameter, accumulated stickiness magnitude (ASM), describing how much discomfort a wetted fabric offered throughout the drying period, was developed. Thin cotton fabrics (fabric W01 and W03), having higher saturation level after contacting with wetted skin surface, arouse stronger stickiness feeling and their ASM is remarkably higher. The difference in stickiness estimates is due to the difference in chemical composition and surface geometry. This study suggests us the way to predict perceived stickiness in fabrics with different wetness levels which is useful for applications like sportswear, intimate apparel or healthcare products.

## 1. Introduction

Human skin having extended contact with textiles, from sleeping, working, walking to exercising, is prone to be susceptible to the condition of clothing material. The presence of sweat or external moisture will wet the clothing, increasing the garment weight and the adhesion between garment and the body, thus resulting in sensorial discomfort and a sticky feel. Stickiness, in terms of friction and surface tension of water, at the skin–textile interface is often associated with the perception of clinginess or clamminess (i.e. the feeling of discomfort). It describes the adhesion of two surfaces, for example, a wet material against the skin. Textile–skin friction is a predominant factor affecting the sensorial comfort of the wearer [1]. With elevated skin moisture level, skin will be softened. It increases the contact area and friction coefficients between fabric and the skin [2–4]. Also, liquid bridges may be formed between the contacting surfaces, increasing the adhesion and friction. Gwosdow *et al*. [3] found that increasing skin wetness is associated with higher fabric–skin friction and subjective displeasure sensation. If the contact pressure and shear force are high or last for prolonged period, it will cause skin irritations, abrasions or other skin injuries [5], for example decubitus [6,7] or friction blisters [8–10].

Sportswear, intimate apparel or healthcare products are inevitably worn under moist conditions. The selection of clothing material becomes important to ensure exercise performance and wear comfort, and textiles which minimize stickiness sensation seem to be an appropriate choice. Objective measures, such as surface friction, surface roughness and water absorbency, are not good predictors for stickiness sensation because several stimuli are contributing to this particular sensation, and it is difficult to isolate all of the relevant variables because of their interaction effects. In order to assess the stickiness sensation perceived in fabrics directly, researchers have developed various subjective assessment methods [11–16]. Assessors were asked to manipulate the samples with their hands in most of the studies (active touch) [11–13,17], whereas samples were put onto assessor's forearms in some experiments (passive touch) [11,14,18]. Or else, wear trials [15,19] or *in vivo* experiments [4,20,21] were performed. So far, researchers have mainly focused on the tactile properties of fabrics in dry condition [3,12,22]. Tang *et al*. [23] found that there is interaction effect between fibre type and moisture content of the fabric on stickiness property, implying that fabrics having good performance in dry state might not perform well in moist condition. Indeed, the chemical composition of the fibres governs the amount and speed of water absorption and so affects the adhesion between the two surfaces [24]. Therefore, the result measured under dry condition cannot be used to predict the stickiness sensation perceived in moist state. Up till now, only few studies have investigated the stickiness sensation perceived in wet fabrics. In Raccuglia *et al*.'s study [11], fabrics were wetted to 50% of total absorption capacity. In Jeon *et al*.'s study [25], fixed amount of water (0.5 and 1.5 ml) was applied to wet the sample. However, they have not investigated the effect of wetness level of fabrics on perceived stickiness systematically. The implication of the amount of water applied to fabric is unclear. Tang *et al*. [14] have also examined the clingy sensation in wetted fabrics. The absolute threshold for clingy sensation is assessed; however, the intensity of perceived clinginess under different wetness levels of fabrics is unknown.

With regard to response measurement technique, psychological scaling approach is usually adopted in assessing the sensorial comfort of textiles [11,12,15,17,26]. The limitations of interval rating scale includes: low involvement felt by the assessor, poor sensitivity, tendency to choose neutral choice and restriction on the use of statistical method [27,28]. In the light of these, psychophysical scaling methods are gaining popularity for assessing wear comfort, and magnitude estimation is one of the examples, where the subject is asked to make numerical estimates of the sensory magnitudes produced by the physical stimuli of known intensities [29,30]. The relationship between sensation magnitude and stimulus magnitude can be described by Stevens' power law (see equation (1.1)). It shows how the subjective magnitude, *ψ*, grows as a power of the stimulus magnitude, *φ*:

1.1 $$\psi =k\varphi^{n}\;\text{or}\;\log_{10}\psi =n\log_{10}\varphi +\log_{10}k,$$

where *k* is a constant that depends on the units of measurement and the exponent, *n*, is the rate of growth of subjective sensation, which differs according to the sensation and testing condition. If *n* = 1, the magnitude estimates grow commensurately with physical intensity. Line length, for example, is governed by an exponent of 1 [31]. If *n* < 1, the magnitude estimates grow slower than physical intensity. The exponents for brightness, moisture sensation in fabric and prickle sensation in fabric are 0.33 [31,32], 0.53 [30] and 0.66 [33], respectively. If *n* > 1, the magnitude estimates grow more rapidly than physical intensity. For example, the exponent for perceived pain of electric shock on the fingertip is 3.5 [31,32]. The major advantages of magnitude estimation include: no boundary to assessor's rating and prevention of the misunderstandings of verbal terms.

In this study, subjective tests were performed to systematically investigate stickiness sensation perceived in fabrics in relation to different wetness levels. The method of magnitude estimation was adopted to quantify the sensation magnitude. Different types of fabrics were investigated to demonstrate the ability of human subjects to differentiate the stickiness properties among samples. This study demonstrates a useful approach when selecting fabrics for functional apparel, sportswear, sock and medical textiles where the wearing condition is stressful. Against the above-mentioned research background, the objectives of this paper are (i) to examine the relationship between perceived stickiness and evaporation time on each fabric type; (ii) to examine the relationship between perceived stickiness and the intensity of moisture stimuli on each fabric type; and (iii) to compare the stickiness estimates among different fabrics. In the current study, we will verify three research hypotheses.

Hypothesis 1: The magnitude estimates of stickiness decrease with evaporation time of fabrics.
Hypothesis 2: The magnitude estimates of stickiness increase with the intensity of moisture stimuli on fabrics.
Hypothesis 3: The magnitude estimates of stickiness vary with fabric types.

## 2. Material and methods

### 2.1. Assessors

Twenty-one healthy assessors were invited for stickiness sensation assessment, of which 13 female and eight male assessors (aged between 23 and 38, average = 28) who have completed the training exercise were confirmed as reliable assessors. They should not have any background knowledge on the samples and not suffer from any skin diseases or peripheral neuropathy.

### 2.2. Experimental condition

All experiments were conducted in a climatic chamber where the temperature was 20 ± 2°C, the relative humidity was 65 ± 5% and air velocity was less than 0.15 m s^−1^.

### 2.3. Stimuli

**Figure 1.**
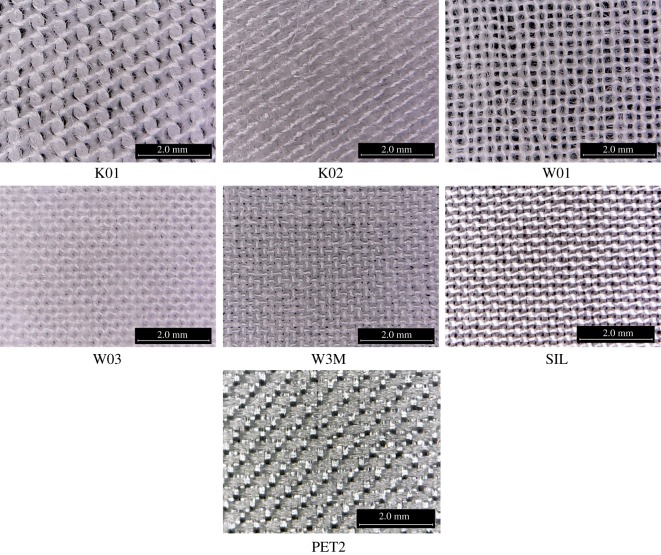
Magnified images of the test fabrics.

Seven types of fabrics, comprising different texture and fibre composition, are selected for subjective stickiness assessment. The specifications of these fabrics are summarized in table 1, whereas the microscopic images of these fabrics are shown in figure 1. Fabrics made by synthetic fibres are hydrophobic, whereas cellulosic ones are hydrophilic. They behave differently when in contact with water and therefore are expected to bring different intensities of stickiness sensation. ‘K01’ and ‘K02’ are regular knitted fabrics for casualwear. ‘W01’ and ‘W03’ are regular shirting material. High-performance polyester ‘W3M’ is intended for sportswear. Polyester satin ‘PET2’, providing glossy and slippery texture, and regular silk ‘SIL’ are intended for sleepwear with luxurious feature.

**Table 1.** Sample specifications.

fabric code	fabric type	fabric structure	fibre content	yarn count (tex)^a^	weight (g m^−2^)	thickness (mm)	porosity	water absorption time (s)^b^	water absorption capacity (WAC) (mg cm^−2^)^c^	surface friction (MIU)^d^	surface roughness (SMD)^d^
K01	knitted	single jersey	60% polyester, 40% cotton	18.4	144.6	0.56	0.8224	15.5	37.17	0.191	1.68
K02	knitted	single jersey	95% rayon, 5% spandex	18.4	259.0	0.86	0.8022	0.7	61.54	0.211	2.26
W01	woven	plain	cotton	7.4	56.6	0.37	0.9002	31.8	14.62	0.177	3.44
W03	woven	plain	cotton	14.8	156.9	0.42	0.7598	13.0	18.26	0.181	2.89
W3M^e^	woven	plain	96% polyester, 4% spandex	6.6	89.1	0.28	0.7685	>60	16.46	0.210	2.36
SIL	woven	plain	silk	5.9	57.9	0.16	0.7299	>60	12.40	0.139	1.89
PET2	woven	satin	polyester	4.5	66.8	0.12	0.6024	>60	7.27	0.132	1.68

^a^Yarn coarseness (the higher the number, the coarser the yarn).

^b^Water absorption time was assessed by wettability test (AATCC 79) [34].

^c^Measured according to Tang *et al*. [35].

^d^Measured by Kawabata Evaluation System for Fabrics (KES-F4).

^e^Fabric manufacturer defined it as high-performance polyester fabric for sportswear application.

These specimens (12 × 12 cm) were conditioned for at least 24 h prior to the assessment. At the onset of subjective assessment, each fabric was wetted according to the standardized procedures mentioned in §2.4 and then dried for different duration (i.e. 0, 16, 32, 48 and 64 min) under standard atmospheric condition (20 ± 1°C, 65 ± 5% RH and wind velocity 0.15 m s^−1^) to achieve different wetness levels. The sample was not re-used so that body grease and other contaminants will not affect the assessment.

### 2.4. Apparatus and experimental protocol

**Figure 2.**
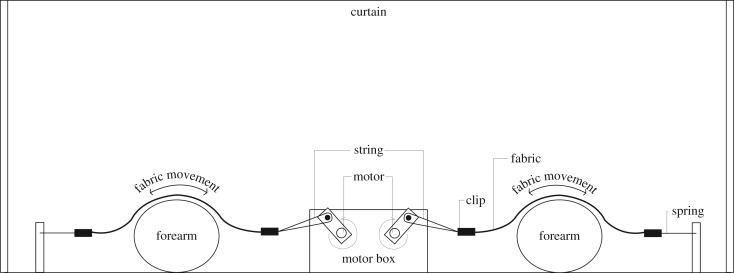
Schematic diagram showing the set-up for subjective stickiness assessment with the use of BMS.

**Figure 3.**
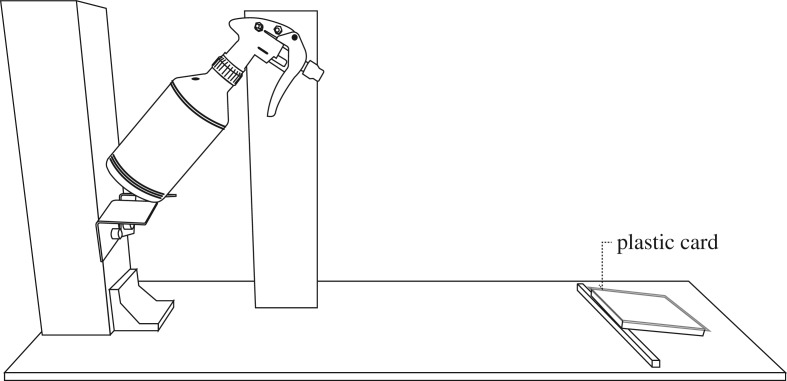
Set-up for applying fixed amount of water to the ‘skin’ (i.e. plastic card).

The experimental protocol was with reference to our previous investigation using the method of magnitude estimation [18]. In brief, the variable stimulus and the reference stimulus were pre-wetted to imitate the sweat-induced fabric surface, so that skin stickiness can be experienced. As shown in figure 2, the variable stimulus was put on one forearm, while the reference stimulus was put on another forearm, allowing pairwise comparison of the samples. These stimuli were moved to-and-fro the volar forearms by the body movement simulator (BMS) automatically to simulate body movement during wear. At the 15th second of contact, the assessor was asked to make magnitude estimation (i.e. assign numerical value) of the variable stimulus relative to the perceived magnitude of the reference stimulus and the modulus (i.e. 100) using a ratio principle. Any positive and non-zero number can be used. If the stickiness sensation seems twice as strong in variable stimulus when compared with reference stimulus, variable stimulus should be rated as 200 (i.e. 2 × 100 = 200). If the variable stimulus is half as strong, it should be rated as 50 [31,36]. Fabric ‘W03’ added with 0.8 g of water, which the panel leader considered its stickiness property to be approximately at the middle of the samples, was chosen as the reference stimulus. The amount of water applied, which was set empirically, does not saturate the reference fabric. According to ISO 11056 [37], the intensity of reference sample should be close to the geometric mean of all samples tested. If the reference presents an extreme value for the attribute, it would induce distortion and reduce the sensitivity of the method. The set-up for water supply unit is illustrated in figure 3.

In order to prepare wet skin surface, fixed amount of water (variable stimulus = 1.4 g (i.e. 9.7 mg cm^−2^); reference stimulus = 0.8 g (i.e. 5.6 mg cm^−2^)) was sprayed onto the plastic card (12 × 12 cm). Subsequently, each sample was put onto the wetted plastic card and pressed at 2.5 g cm^−2^ pressure for 5 s to absorb the ‘sweat’. The pressure was set with reference to the gravimetric absorbency testing system (GATS) [38] to achieve good contact between fabric and wetted plastic card. This assumes that sweating rate is fixed no matter what clothes are worn, which allows fair comparison between fabrics. The wetted samples were then dried for different durations (i.e. 0, 16, 32, 48 and 64 min). After drying for predetermined duration, the sample was picked up from the plastic card and the mass of water added to each sample was recorded, so the intensity of the moisture stimuli were known.

For each subject, all samples were presented once, so 35 pairs (seven fabric types × five wetness levels) of specimens were assessed. To prevent sensory fatigue, there will be at least 45 s resting period between each sample. During that time, the assessor should use soft tissue paper to gently remove the residual water from skin surface. The actual testing lasted for 75 min normally and in some cases may extend to 90 min. Once feeling uncomfortable, the assessor could ask for rest or even terminate the assessment.

### 2.5. Data rescaling and statistical analysis

Data collected from the assessor panel were rescaled by the total rescaling method according to ISO 11056 [37] and ASTM E1697 [36]. The reason for data rescaling is that different numerical scales were used by the assessors which may produce a significant assessor effect. After rescaling, data will be in logarithmic (log_10_) scale and the total magnitude of the response for the 35 samples should be identical for each assessor. Data then underwent further statistical analysis using SPSS 22. The significance level of the statistical analyses conducted in this study was set at 0.05. Pearson correlation coefficient was performed to study the within-subject reliability. Kendall's coefficient of concordance (*W*), a measure of agreement among judges, ranging from 0 (no agreement) to 1 (complete agreement), was used to study the between-subject consistency. Repeated measures analysis of variance (ANOVA) was used to examine the between-sample differences. Meanwhile, Friedman test, a non-parametric equivalent of a one-sample repeated measures design, was used to test the null hypothesis that *k*-related variables come from the same population and was adopted to test the between-fabric difference in terms of the ranking of how sticky the samples are. Additionally, paired *t*-tests were used to pinpoint which fabrics, in particular, have significant differences against another.

## 3. Results and discussions

### 3.1. Within-subject reliability

The within-subject reliability is examined by looking at the correlation between sensation magnitude (i.e. magnitude estimates of stickiness) and intensity of the physical stimuli (i.e. amount of water present in fabric) for each assessor on each fabric type. The results for the correlation analysis are shown in table 2. Positive Pearson correlation coefficient suggests a positive linear relationship between two variables. Negative Pearson correlation coefficient implies that more water present in fabric does not associate with stronger stickiness sensation. This further suggests that the sensory acuity of the assessor might not be strong enough to detect the differences among fabrics with different wetness levels. As seen from table 2, Pearson correlation coefficient of more than 90% of the cases is positive, implying that the majority of the assessors are reliable. However, after collecting all data, assessor #18 reported that he has suffered from diabetes. Researches have proved that diabetic patients have poorer sensory acuity in terms of thermal sensation [39–41]. According to assessor #18's physical condition, all of his data are rejected for further analysis.

**Table 2.** The Pearson correlation coefficient between normalized magnitude estimates of stickiness and the amount of water present in fabric for each assessor on each fabric type (*p*-value of the correlation analysis shown within the bracket).

fabric code	assessor no.																				
	1	2	3	4	5	6	7	8	9	10	11	12	13	14	15	16	17	18	19	20	21
K01	0.79 (0.11)	0.50 (0.40)	0.37 (0.54)	−0.46 (0.44)	0.29 (0.64)	0.91 (0.03)	0.89 (0.04)	0.37 (0.55)	0.09 (0.89)	−0.03 (0.96)	0.10 (0.87)	0.65 (0.24)	0.44 (0.46)	0.70 (0.19)	0.76 (0.13)	0.94 (0.02)	0.57 (0.32)	0.18 (0.78)	0.56 (0.32)	−0.37 (0.54)	0.45 (0.44)
K02	−0.60 (0.29)	0.69 (0.20)	0.47 (0.43)	0.05 (0.93)	0.83 (0.09)	0.21 (0.73)	−0.34 (0.58)	−0.57 (0.31)	−0.27 (0.67)	0.48 (0.41)	0.95 (0.01)	0.07 (0.91)	0.34 (0.57)	0.32 (0.59)	0.82 (0.09)	0.99 (0.00)	0.47 (0.42)	0.58 (0.30)	−0.40 (0.51)	0.72 (0.17)	0.42 (0.49)
W01	0.89 (0.04)	0.80 (0.11)	0.78 (0.12)	0.95 (0.01)	0.90 (0.04)	0.66 (0.22)	0.87 (0.05)	0.89 (0.05)	0.84 (0.08)	0.95 (0.01)	0.97 (0.01)	0.76 (0.13)	0.83 (0.08)	0.85 (0.07)	0.90 (0.04)	0.75 (0.15)	0.81 (0.10)	0.75 (0.14)	0.85 (0.07)	0.85 (0.07)	0.90 (0.04)
W03	0.58 (0.31)	0.61 (0.27)	0.23 (0.71)	0.82 (0.09)	0.52 (0.37)	0.89 (0.04)	0.98 (0.00)	0.56 (0.33)	0.77 (0.13)	0.77 (0.13)	0.89 (0.04)	0.97 (0.01)	0.69 (0.20)	0.84 (0.08)	0.75 (0.14)	0.72 (0.17)	0.59 (0.30)	−0.90 (0.04)	0.47 (0.43)	0.59 (0.29)	0.17 (0.79)
W3M	0.85 (0.07)	0.59 (0.30)	0.66 (0.22)	0.22 (0.72)	−0.28 (0.66)	−0.03 (0.96)	0.42 (0.48)	0.95 (0.01)	0.57 (0.31)	0.72 (0.17)	0.24 (0.69)	0.44 (0.46)	0.75 (0.14)	0.40 (0.51)	0.41 (0.49)	−0.07 (0.91)	0.20 (0.75)	0.65 (0.24)	0.70 (0.19)	0.35 (0.56)	0.32 (0.60)
SIL	0.82 (0.09)	0.81 (0.10)	0.79 (0.12)	0.74 (0.15)	0.85 (0.07)	0.96 (0.01)	0.79 (0.11)	0.97 (0.01)	0.79 (0.11)	0.66 (0.22)	0.93 (0.02)	0.93 (0.02)	0.73 (0.16)	0.71 (0.18)	0.70 (0.19)	0.71 (0.18)	0.76 (0.13)	0.45 (0.44)	0.74 (0.15)	0.13 (0.84)	0.78 (0.12)
PET2	0.93 (0.02)	0.89 (0.05)	0.92 (0.03)	0.85 (0.07)	−0.49 (0.40)	0.95 (0.01)	0.79 (0.11)	0.81 (0.10)	0.89 (0.04)	0.68 (0.20)	0.89 (0.04)	0.91 (0.03)	0.82 (0.09)	0.97 (0.01)	0.89 (0.04)	0.78 (0.12)	0.87 (0.06)	0.87 (0.05)	0.69 (0.20)	0.39 (0.52)	0.90 (0.04)

### 3.2. Between-subject consistency

After confirming the reliability of individual assessor, this part aims to check the consistency between different assessors. Kendall's coefficient of concordance was used to describe the level of agreement in ranking the 35 samples between assessors. Kendall's coefficient of concordance is 0.576 (*p* = 0.000 < 0.05) which is fairly high, indicating fairly good agreement between the assessors.

### 3.3. Between-fabric difference

Two-way repeated measures ANOVA test was performed with ‘fabric’ and ‘evaporation duration’ being the independent variables. This part aims to check if there are any significant differences among the seven fabric types and among the five evaporation durations in terms of the normalized magnitude estimation of stickiness. The results show that there are significant overall differences in the stickiness estimates for the seven fabric types [Greenhouse–Geisser *F* = 25.077, *p* = 0.000 < 0.05] and the five evaporation durations [Greenhouse–Geisser *F* = 43.591, *p* = 0.000 < 0.05].

The non-parametric Friedman test examines the null hypothesis that the normalized magnitude estimates of stickiness are the same for the 35 fabrics in terms of its ranking. The results indicate that the Friedman *χ*^2^ statistics are significant, *χ*^2^ (d.f. = 34) = 391.793, *p* = 0.000 < 0.05. Thus, it can be concluded that there are significant between-fabric differences.

**Figure 4.**
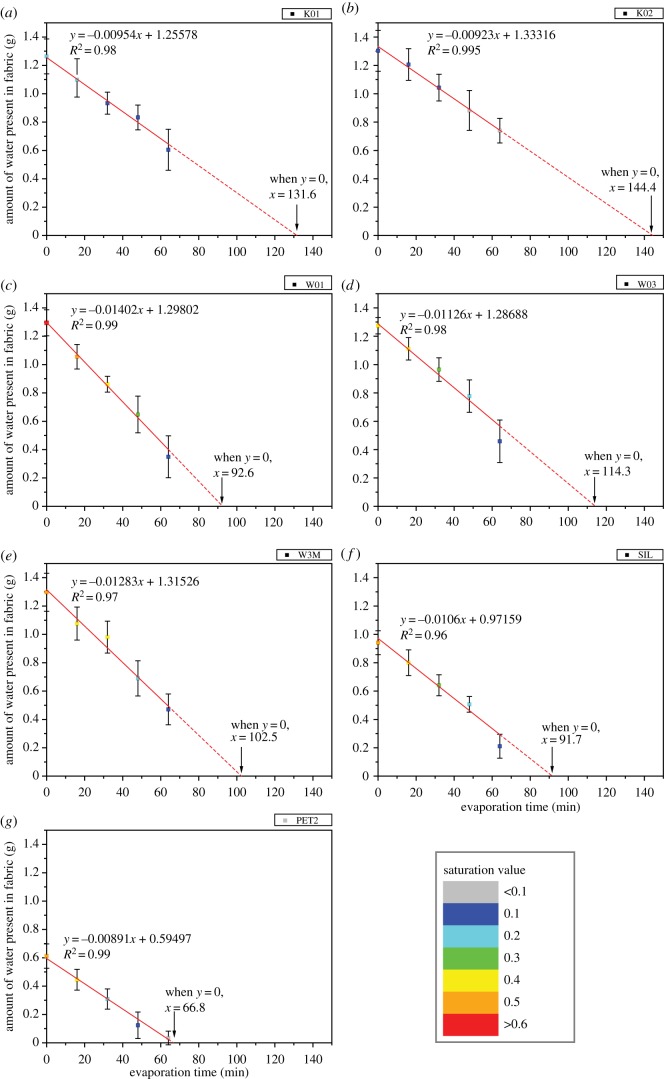
Plots of amount of water present in fabric at different evaporation time. Error bars denote one standard deviation of uncertainty. The colour of data points denotes the saturation value of the fabric. The solid line shows that the points are fitted with linear function, whereas the dash line shows the extrapolation from the trend line. The *x*-intercept is the projected TDT.

Repeated measures ANOVA test and Friedman test state whether there are overall differences, but do not report which pairs have significant differences. Therefore, paired *t*-test was performed to study the effect of evaporation duration within each fabric type and the results are summarized in table 3. It shows that the stickiness estimates for fabric ‘K02’ do not have significant difference under different evaporation durations (*p* > 0.05). This is because the water absorbency of fabric ‘K02’ is good, but the amount of water evaporated is comparatively low. As seen from figure 4, only 0.56 g of water has been evaporated even dried for 64 min (in comparison, 0.95 g of water evaporated in thin cotton fabric ‘W01’). For a thick hydrophilic fabric like ‘K02’, the added water might be bound within the inter-yarn or inter-fibre space; consequently, it is not easy to distinguish the difference in stimulus magnitude. For fabric ‘W3M’ dried with different durations, the between-fabric difference can hardly be sensed as well (in one out of the 10 pairs only). In brief, it shows that the effect of evaporation duration on stickiness estimates is not prominent for fabrics ‘K02’ and ‘W3M’. However, it is significant for fabrics ‘W01’, ‘W03’, ‘SIL’ and ‘PET2’.

**Table 3.** Pairwise comparison matrix indicating *p*-value of each pair (fabrics with different evaporation durations were compared).

	effect of evaporation duration																																		
	K01					K02					W01					W03					W3M					SIL					PET2				
(min)	64	48	32	16	0	64	48	32	16	0	64	48	32	16	0	64	48	32	16	0	64	48	32	16	0	64	48	32	16	0	64	48	32	16	0
64		0.128	0.007	0.005	0.002		0.900	0.568	0.099	0.214		0.007	0.000	0.000	0.000		0.046	0.011	0.004	0.000		0.508	0.134	0.099	0.000		0.002	0.000	0.000	0.000		0.009	0.000	0.000	0.000
48			0.955	0.742	0.069			0.512	0.094	0.171			0.014	0.000	0.000			0.386	0.001	0.000			0.555	0.162	0.015			0.100	0.017	0.000			0.031	0.023	0.000
32				0.601	0.036				0.106	0.324				0.002	0.000				0.057	0.000				0.569	0.048				0.069	0.000				0.947	0.000
16					0.136					0.799					0.005					0.000					0.168					0.000					0.000
0

Paired *t*-test was also performed to study the effect of fabric type within each evaporation duration and the results are summarized in table 4. When the evaporation duration is 64 min, significant differences are found in four out of the 21 pairs only, while 16 pairs are found when the evaporation duration is 16 and 0 min. This suggests that between-fabric difference is commonly found when the evaporation time is shorter (i.e. at the onset of recovery period). With increasing evaporation time (i.e. after long period of recovery), the between-fabric difference can hardly be detected. This is because most of the free water from the fabric surface has been evaporated and the amount of water that remained is remarkably low in thin fabrics (e.g. SIL, PET2) or water stayed mainly in the internal part of those thick fabric (e.g. K01, K02). Therefore, it is hard to detect the difference in stickiness estimates after prolonged recovery period.

**Table 4.** Pairwise comparison matrix indicating *p*-value of each pair (different fabric types were compared under same evaporation duration).

	effect of fabric type																																		
	64 min							48 min							32 min							16 min							0 min						
	K01	K02	W01	W03	W3M	SIL	PET2	K01	K02	W01	W03	W3M	SIL	PET2	K01	K02	W01	W03	W3M	SIL	PET2	K01	K02	W01	W03	W3M	SIL	PET2	K01	K02	W01	W03	W3M	SIL	PET2
K01		0.997	0.023	0.740	0.553	0.031	0.463		0.154	0.023	0.308	0.391	0.015	0.215		0.030	0.000	0.050	0.529	0.000	0.000		0.473	0.000	0.004	0.751	0.000	0.000		0.043	0.000	0.000	0.588	0.000	0.000
K02			0.060	0.716	0.627	0.089	0.459			0.001	0.006	0.191	0.000	0.003			0.000	0.001	0.096	0.000	0.000			0.000	0.000	0.467	0.000	0.000			0.000	0.000	0.018	0.000	0.000
W01				0.257	0.036	0.789	0.028				0.019	0.000	0.150	0.760				0.036	0.000	0.617	0.786				0.001	0.000	0.033	0.029				0.000	0.000	0.820	0.107
W03					0.989	0.302	0.289					0.019	0.002	0.466					0.008	0.008	0.001					0.002	0.002	0.082					0.000	0.001	0.089
W3M						0.103	0.301						0.000	0.034						0.000	0.000						0.000	0.001						0.000	0.000
SIL							0.057							0.403							0.297							0.532							0.068
PET2																																			

### 3.4. Amount of water present in fabric as a function of evaporation time

In figure 4, the drying performance of fabrics can be interpreted. The *y*-axis of the plots represents the amount of water present in fabric before it is delivered onto the assessor's forearm. It describes the intensity of moisture stimulus. The *x*-axis is the evaporation duration. The colour of each data point denotes its saturation value which is calculated by dividing the amount of water present in fabric by its water absorption capacity (WAC). These plots show that the amount of water present in fabric and its saturation value decreases with evaporation duration. Also, the drying behaviour varies with fabric type. The points in each plot are fitted with linear function. The slope, *y*-intercept, *x*-intercept, coefficient of determination (*R*^2^) and saturation value are shown in each plot correspondingly.

First, the slope represents the drying rate of fabrics. Among the seven fabric types, the slope of fabric ‘W01’ is the steepest (−0.01402), followed by ‘W3M’ (−0.01283), ‘W03’ (−0.01126), ‘SIL’ (−0.0106), ‘K01’ (−0.00954), ‘K02’ (−0.00923), whereas the slope for ‘PET2’ is the gentlest (−0.00891). The lowest drying rate for fabric ‘PET2’ is due to its poor transplanar wicking property. Water stayed mainly at the back side of fabric (i.e. next to the plastic card), but does not transfer to its face side, so only little water vapour can transport through the fabric. For fabric ‘K01’ and ‘K02’, they are relatively thick fabrics. The water spreading area in thick fabrics is smaller in general, resulting in smaller exposed surface for water evaporation [42]. Therefore, the drying rate of fabric ‘K01’ and ‘K02’ is moderate. On the other hand, the thickness of fabric ‘W01’ is low and its porosity is high, as shown in table 1 and figure 1, respectively. Its in-plane and transplanar wicking is good and free water may present over its pores. This facilitates water evaporation and so its drying rate is high.

Second, the *y*-intercept shows the amount of water present in fabric after putting them onto the wetted plastic card (i.e. evaporation time = 0 min). The more water present, the higher the absorbency. Figure 4 shows that the absorbency of fabric ‘K02’ is the highest (1.333), followed by ‘W3M’ (1.315), ‘W01’ (1.298), ‘W03’ (1.287), ‘K01’ (1.256), ‘SIL’ (0.972) and ‘PET2’ (0.595). The higher absorbency for fabric ‘K02’ is because it is a thick rayon fabric and rayon has a high number of hydroxyl groups along the polymer chains which support water absorption [43]. Among these seven fabrics, the absorbency of fabric ‘SIL’ and ‘PET2’ is significantly lower. Less than 1 g of water was absorbed by these fabrics which means that their absorbency is much lower than the water supplied and there is residual water left on the plastic card. During actual wear situation, this residual water may roll off the skin surface and so these fabrics were discarded for further analysis.

Third, the *x*-intercept is extrapolated from the trend line by fitting *y* = 0 to the linear equation. When *y* = 0, it implies that there is no more water present in fabric and it can be defined as total drying time (TDT). TDT projects the time required to dry the fabric completely and it assumes that the moisture regain of fabric is zero. Given that the external environmental condition for testing is the same, the TDT is, in fact, affected by many factors, like fabric geometry, the amount of water originally held in the sample [44,45], the moisture distribution within the fabric and area of exposed surface [46]. For fabric ‘PET2’, only 66.8 min is needed to dry the fabric, whereas 144.4 min is required to dry the fabric ‘K02’.

Fourth, the *R*^2^ of each plot is higher than 0.95. This implies that the amount of water present in fabric and evaporation time are linearly related, implying that water evaporated from the fabric at a constant rate.

Fifth, additional information can be obtained from figure 4. That is saturation value of fabrics. It ranges from 0.033 (PET2 dried for 64 min) to 0.614 (W01 dried for 0 min). After drying for 64 min, the reduction in saturation value is dramatic in fabric ‘PET2’ (data points change from orange to grey) and ‘W01’ (data points change from red to navy), whereas the reduction in fabric ‘K01’ and ‘K02’ is minor.

### 3.5. Magnitude estimates of stickiness as a function of evaporation time

When one stops exercising and begins to rest, active sweating might cease shortly, allowing the skin and clothing layers to eventually dry [45]. This section aims to investigate the perceived stickiness in the recovery period after exercising and sweating. In order to simplify the experiment, there are four assumptions. First, it assumes that there is no further sweat secretion during the onset of the recovery period. Second, drying rate at room temperature is in proportion to the drying rate at skin temperature. Third, the sweating amount is independent of fabric type (i.e. 1.4 g of water is applied to all fabric types). Fourth, the stickiness sensation is negligible when the fabric is completely dry.

**Figure 5.**
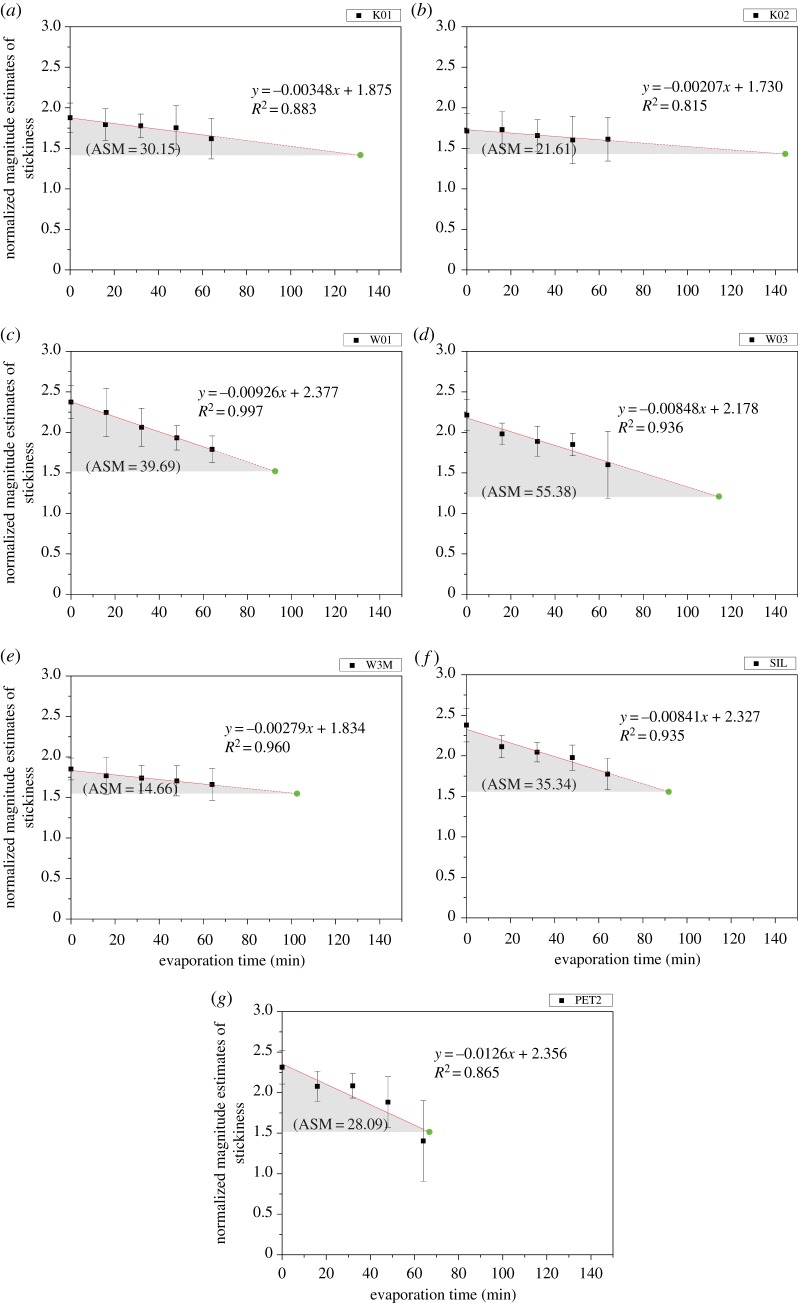
Normalized magnitude estimates of stickiness perceived in fabrics against evaporation time. Each data point represents the average response from 20 assessors. Error bars denote one standard deviation of uncertainty. The solid line shows that the points are fitted with linear function, whereas the dash line shows the extrapolation from the trend line. The green circles show the perceived stickiness at TDT (i.e. when fabric is dry). The grey area denotes the degree of discomfort that the wearer has suffered before the fabric becomes dry.

The semi-log plot, as illustrated in figure 5, shows that normalized magnitude estimates of stickiness and evaporation time is negatively related. Equations for the relation were calculated according to the method of least square and strong relationship is observed. The *R*^2^ ranges from 0.815 to 0.997 which is very high. The linear regression helps to estimate the perceived stickiness under different evaporation time. It can be done simply by substituting any evaporation time into the linear regression.

The trend line is then extrapolated to the time when fabric dries completely (i.e. *x* = TDT value obtained from figure 4). This indicates the stickiness estimates of a dry fabric. By calculating the area underneath the linear regression (from the time when the recovery period starts to the time when fabric dries completely) and subtracting the stickiness estimates caused by dry fabric, it describes how much discomfort (i.e. the degree and duration of discomfort) the wearer has suffered caused by sweating. This area, termed as accumulated stickiness magnitude (ASM), is triangular and is marked in grey in figure 5. The calculation of this area is shown in equations (3.1)–(3.3). ASM is, in fact, varied by drying rate of fabric apart from the magnitude of stickiness sensation. Higher ASM implies poor wear comfort. As shown in figure 5, the ASM for fabric ‘W03’ is the highest, followed by ‘W01’, ‘SIL’, whereas the ASM for ‘W3M’ is the lowest.

3.1 $$\text{ASM}=\int_{0}^{\mathrm{TDT}}\left(mx+c)dx-[m(\text{TDT})+c](\text{TDT}\right),$$

3.2 $$\text{ASM}=\left[\frac{m}{2}x^{2}+cx\right]\begin{array}{c} \text{TDT}\\ 0 \end{array}-[m{\left(\text{TDT}\right)}^{2}+c(\text{TDT})]$$

3.3 $$\;\text{and}\;\text{ASM}=-\frac{1}{2}m{\left(\text{TDT}\right)}^{2}.$$

### 3.6. Magnitude estimates of stickiness as a function of amount of water present in fabric

**Figure 6.**
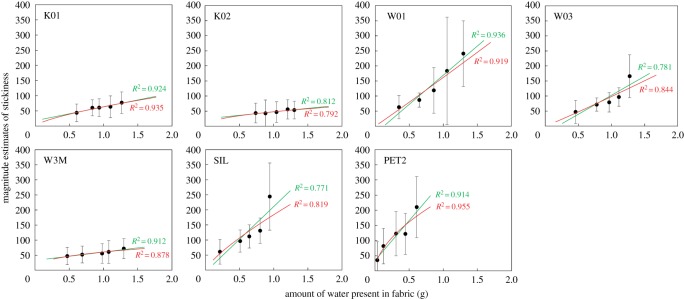
Magnitude estimates of stickiness as a function of amount of water present in the fabric. The green line shows the linear function for data points, whereas the red line shows the power function.

This section aims to investigate the stickiness sensation perceived in fabrics with different moisture levels, from mildly wet to completely wet condition. For ease of presentation, the normalized magnitude estimate data were transformed with antilogarithm. The relationship between sensation magnitude and stimulus intensity is examined and is illustrated in figure 6. Data points are fitted with linear and power function. The green lines denote the linear function, while the red line shows the power function. Power function relationship is evident in four out of the seven fabric types (i.e. K01, W03, SIL and PET2). For the seven fabric types, the average *R*^2^ for the linear function is 0.86, while the average *R*^2^ for the power function is 0.88. Although power function relationship is only little more evident, power function has demonstrated ample applicability on other sensory modalities [47] and so it was chosen for further analysis.

**Figure 7.**
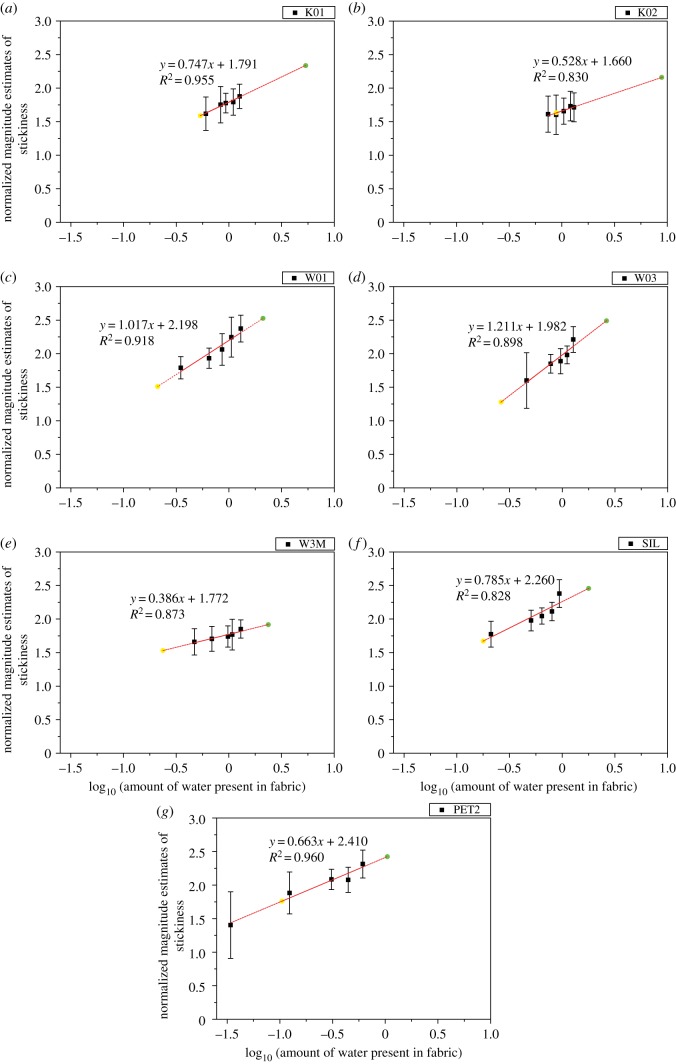
Log–log plots showing normalized magnitude estimates of stickiness (*ψ*) against moisture stimuli (*φ*). Error bars denote one standard deviation of uncertainty. The solid line shows that the points are fitted with linear function, whereas the dash line shows the extrapolation from the trend line. The yellow circles show the perceived stickiness at 10% WAC, whereas the green circles show the perceived stickiness at 100% WAC.

Next, the magnitude estimates of stickiness (*ψ*) were plotted against the amount of water present in fabric (*φ*) in log–log coordinates (figure 7). The method of least squares was used to find the linear regression that best fit the data. A log–linear relationship was found that supports Stevens' power law [28], *ψ* = *kφ^n^*, where the magnitude of perceived stickiness (*ψ*) increases as a power function (*n*) of the stimulus magnitude (*φ*). Wetter fabrics are associated with stronger stickiness estimates because moisture from fabric will hydrate and soften the skin. Liquid bridges might be formed between the contacting surfaces which increase the contact area, adhesion force and friction [48]. Researchers have reported that clothing is judged more comfortable if there are fewer contact points between fabric and skin surface and the skin surface is dry [49].

The statistical analysis results, including the linear regression, power regression and *R*^2^, are summarized in table 5. Among the seven fabric types, the coefficients of determination range from 0.828 to 0.960 indicating that over 82.8% of the change in perceived stickiness was accounted for by the increase in the amount of water in fabric.

**Table 5.** Linear regression, power regression, exponent and coefficient of determination obtained when judging the stickiness sensation of fabrics with different moisture levels.

	linear regression	power regression			coefficient of determination
		equation	exponent (*n*)	constant (*k*)	
K01	log(*Ψ*) = 0.747 log(*φ*) + 1.791	*Ψ* = 61.80*φ*^0.747^	0.747	61.80	*R*^2^ = 0.955
K02	log(*Ψ*) = 0.528 log(*φ*) + 1.660	*Ψ* = 45.71*φ*^0.528^	0.528	45.71	*R*^2^ = 0.830
W01	log(*Ψ*) = 1.017 log(*φ*) + 2.198	*Ψ* = 157.76*φ*^1.017^	1.017	157.76	*R*^2^ = 0.918
W03	log(*Ψ*) = 1.211 log(*φ*) + 1.982	*Ψ* = 95.94*φ*^1.211^	1.211	95.94	*R*^2^ = 0.898
W3M	log(*Ψ*) = 0.386 log(*φ*) + 1.772	*Ψ* = 59.16*φ*^0.386^	0.386	59.16	*R*^2^ = 0.873
SIL	log(*Ψ*) = 0.785 log(*φ*) + 2.260	*Ψ* = 181.97*φ*^0.785^	0.785	181.97	*R*^2^ = 0.828
PET2	log(*Ψ*) = 0.663 log(*φ*) + 2.410	*Ψ* = 257.04*φ*^0.663^	0.663	257.04	*R*^2^ = 0.960

The exponent from the power regression represents the rate of growth of subjective stickiness sensation produced by fabric's wetness level. The exponents found in this study range from 0.386 for fabric ‘W3M’ to 1.211 for fabric ‘W03’. For high-performance polyester fabric ‘W3M’, if the magnitude of moisture stimulus were increased by one logarithmic unit, the corresponding increase in stickiness estimates would only be 0.385 expressed in logarithmic units. Thus, stickiness sensation grows slowly as moisture stimulus intensity is increased. This is a favourite feature and can attribute to excellent water transport property of fabric ‘W3M’. Odour intensity, loudness and brightness, for example, are governed by exponents less than 1 [31]. However, for fabric ‘W03’, its exponent is 1.211. It means that the perceived stickiness increased approximately 1.2 times per unit change in moisture intensity and implies that small changes in the intensity of moisture stimulus produced dramatic changes in the psychological continuum of perceived stickiness as quantitatively expressed by Stevens' power exponent, *n*. For fabric ‘W01’, the exponent is approximate to 1, meaning that the magnitude estimates grows commensurately with physical intensity. These suggest that the effect of moisture is notable in these ordinary shirting materials (W01 and W03).

The constant from the power regression also provides meaningful data. It indicates the stickiness estimates when log *φ* = 0 (*φ* = 1 g of water present in fabric). As shown in table 5, the constant ranges from 45.71 (K02) to 257.04 (PET2). It means that when there is 1 g of water in fabric, the perceived stickiness for fabric ‘PET2’ is remarkably higher. Fabric ‘K01’ and ‘K02’ are intended for casualwear application, the stickiness estimates for fabric ‘K02’ are much lower under this wetness condition and so it is preferred. On the other hand, fabric ‘W01’ and ‘W03’ are intended for shirting material, the stickiness estimates for fabric ‘W03’ is much lower under this wetness condition and this can attribute to higher water absorption capacity for fabric ‘W03’. For the application of sleepwear, the stickiness estimates for fabric ‘SIL’ are much lower than those of ‘PET2’ under this wetness condition and this suggests that fabric ‘SIL’ is recommended if the sleepwear is intended to be worn in hot condition.

In figure 7, each trend line is extrapolated to log *φ* = 10% of WAC (i.e. yellow points) and log *φ* = 100% of WAC (i.e. green points). Therefore, the perceived stickiness under different wetness levels can be estimated.

### 3.7. Limitations of the study

First, an assumed fixed sweating rate ensures fair comparison between fabrics, but it should be noted that fabric type may inherently affect the sweating rates and so fixed sweating rate may not fully reflect the actual wear condition. However, only time-consuming wear trial can overcome this problem. Second, fabrics were dried at flat surface and room temperature. It is assumed that this is in proportion to the drying condition at skin surface and skin temperature. Third, skin wetness and temperature are assumed to return to a constant level after 45 s resting period.

## 4. Conclusion

In this study, seven types of fabrics were wetted by contacting a simulated skin surface with a fixed amount of sweat and dried for different durations, resulting in different wetness levels. After preparing fabrics with different wetness levels, its stickiness estimates were assessed using the method of magnitude estimation. The wet fabrics (variable and reference stimuli) were moved against assessors' volar forearms automatically by BMS. BMS provides repeatable fabric movement and allows real-time comparison between variable and reference stimuli, so ensuring testing reproducibility.

The normalized magnitude estimates of stickiness against different evaporation time, implying the stickiness sensation perceived during the recovery period, were studied and fitted with linear function. The slope of the fitted line represents the rate of change in stickiness estimates during the recovery period. Negative slope was observed in all fabric types studied. The trend line is then extrapolated to the time when fabric dries completely (i.e. *x* = TDT). This tells us the ultimate stickiness estimates when the fabric is dry. A newly developed parameter, called ASM, describes how much stickiness discomfort the wearer has suffered during the recovery period (i.e. from wet to dry condition). Higher ASM implies poor wear comfort and the ASM for thin cotton fabrics (W03, W01) is comparatively high.

Additionally, the magnitude estimates of stickiness were found to be a power function to the amount of water present in fabrics and this can be described by Stevens' power law. The exponent from the power regression describes the growth rate of perceived stickiness produced by the amount of water present in the fabric. The exponents range from 0.386 (high-performance polyester fabric, W3M) to 1.211 (ordinary cotton woven fabric, W03). When the exponent is low, it implies that stickiness sensation grows slowly as moisture stimulus intensity increases. Besides, the constant value from the power regression tells us the perceived stickiness when there is 1 g of water present in the sample.

This paper demonstrates the possibility for a group of assessors to discriminate different fabrics with different wetness levels on the basis of the stickiness interaction with the human forearm. The trend is that stickiness perception increases with wetness level of fabrics and decreases with evaporation time, so the three research hypotheses were accepted. The experimental protocol provides efficient guidelines for researchers and product developers to study stickiness sensation perceived in wet fabrics systematically. The results show that thin cotton fabrics (W01 and W03) in particular give stickier feeling. This study also suggests that magnitude estimation can be used to investigate other sensorial comfort factors where there are direct physical stimuli for correlation analysis.

## Data accessibility

All experimental data are reported in tables 1–5 and figures 4–7. This study has no additional data.
